# Vpr driving DNA methylation variation of CD4 + T cells in HIV-1 infection

**DOI:** 10.1186/s12985-024-02363-5

**Published:** 2024-04-26

**Authors:** Peipei Wang, Zhuoyue Meng, Kai Deng, Zhiliang Gao, Jinfeng Cai

**Affiliations:** 1 Department of Infectious Diseases, The Third Affiliated Hospital, Sun Yat-sen University, Guangzhou, China; 2 Institute of Human Virology, Key Laboratory of Tropical Disease Control of Ministry of Education, Zhongshan School of Medicine, Sun Yat-sen University, Guangzhou, China; 3 Department of Immunology and Microbiology, Zhongshan School of Medicine, Sun Yat-sen University, Guangzhou, China

**Keywords:** HIV-1, Accessory protein Vpr, DNA methylation, WGBS

## Abstract

**Background:**

Despite the existence of available therapeutic interventions for HIV-1, this virus remains a significant global threat, leading to substantial morbidity and mortality. Within HIV-1-infected cells, the accessory viral protein r (Vpr) exerts control over diverse biological processes, including cell cycle progression, DNA repair, and apoptosis. The regulation of gene expression through DNA methylation plays a crucial role in physiological processes, exerting its influence without altering the underlying DNA sequence. However, a thorough examination of the impact of Vpr on DNA methylation in human CD4 + T cells has not been conducted.

**Methods:**

In this study, we employed base-resolution whole-genome bisulfite sequencing (WGBS), real-time quantitative RCR and western blot to explore the effect of Vpr on DNA methylation of host cells under HIV-1 infection.

**Results:**

We observed that HIV-1 infection leads to elevated levels of global DNA methylation in primary CD4 + T cells. Specifically, Vpr induces significant modifications in DNA methylation patterns, particularly affecting regions within promoters and gene bodies. These alterations notably influence genes related to immune-related pathways and olfactory receptor activity. Moreover, Vpr demonstrates a distinct ability to diminish the levels of methylation in histone genes.

**Conclusions:**

These findings emphasize the significant involvement of Vpr in regulating transcription through the modulation of DNA methylation patterns. Together, the results of this investigation will considerably enhance our understanding of the influence of HIV-1 Vpr on the DNA methylation of host cells, offer potential avenues for the development of more effective treatments.

**Supplementary Information:**

The online version contains supplementary material available at 10.1186/s12985-024-02363-5.

## Background

With an estimated global population of approximately 38 million individuals living with HIV-1, along with an additional 1.5 million new global infections occurring annually [1], it is noteworthy that HIV-1 primarily targets CD4 + T cells, leading to their depletion and ultimately culminating in the development of AIDS and subsequent mortality. Although antiretroviral therapy effectively inhibits HIV-1 replication, it is incapable of completely eradicating the infection due to the persistence of the virus within a restricted subset of latently infected cells. This viral persistence poses a potential risk of reactivating the infection upon discontinuation of treatment [2, 3]. Consequently, there exist considerable impediments to the prevention and treatment of HIV-1 [4]. The intricate interplay between HIV-1 and host target cells constitutes a fundamental cause for these formidable hurdles, yet the underlying mechanisms of this interaction remain inadequately comprehended. DNA methylation assumes a pivotal function in the regulation of gene expression without modifying the DNA sequence [5], predominantly manifesting at the carbon-5 position of cytosine residues within a cytosine-guanine dinucleotide (CpG site) [6, 7]. However, numerous diseases have been linked to abnormal methylation patterns [8–11]. Various studies have demonstrated that viral infections can trigger aberrant methylation patterns within the genome of the host [12–14]. Conversely, the integrated proviral genome is also influenced by the epigenetic milieu of the host [12, 15, 16]. Consequently, the interaction between the virus and the host leads to a modified epigenetic environment that impacts both the virus and the infected host cell. Multiple studies offer substantiation that HIV-1 infection elicits alterations in DNA methylation of host cells [17, 18]. DNA methylation is established by a group of enzymes known as DNA methyltransferases (DNMTs), specifically DNMT1, DNMT3A, and DNMT3B [19, 20]. Previous studies have demonstrated that early expressed HIV-1 proteins, such as Tat, Nef, and Rev, can promote the expression of DNMTs by enhancing the activity of the DNMT1 promoter [21–23]. Additionally, Methyl CpG-binding protein-2 (MeCP2), a crucial multifunctional epigenetic regulator, plays a role in recognizing methylated CpG sites and influencing transcription and chromatin structure [24–25]. It has been observed that HIV Tat can induce the expression of miR-132, which subsequently leads to the down-regulation of MeCP2 expression [26]. However, there is limited literature available regarding the impact of other HIV accessory proteins on the methylation of host cell DNA.

Vpr, an accessory protein of HIV-1, warrants investigation due to its incorporation into viral particles and involvement in the early stages of infection. Previous research has demonstrated that Vpr can induce cell cycle arrest during the G2/M phase, facilitate apoptosis of target cells, enhance the expression of HIV-1 genes, disrupt crucial signaling pathways, and counteract host restriction factors within target cells [27–32]. It is hypothesized that Vpr operates by enlisting host target proteins to the DCAF1-DDB1-Cul4 E3 ubiquitin ligase complex [33–34]. Furthermore, recent research has demonstrated that Vpr elicits comprehensive changes in the transcriptomic and proteomic profiles during the initial phases of HIV-1 infection [35–36]. These findings strongly suggest that Vpr exerts a substantial influence on the gene expression patterns of primary CD4 + T cells during HIV-1 infection. Notably, the Ten-eleven translocation methylcytosine dioxygenase (TET) enzymes, which function as DNA demethylase enzymes, catalyze the conversion of 5-methyl-cytosine into 5-hydroxymethyl-cytosine, serving as the initial step in cytosine demethylation. Recent investigations have revealed that HIV-1 Vpr enhances HIV-1 replication by degrading TET2 in macrophages [34, 37]. However, there is a limited amount of research available regarding the impact of HIV-1 infection on DNA methylation within the host genome. Additionally, no studies have been conducted to investigate the effects of Vpr on alterations in the entire genome of CD4 + T cells in terms of DNA methylation.

In this study, we performed base-resolution whole-genome bisulfite sequencing (WGBS) of primary CD4 + T cells with either HIV-1-Vpr or HIV-1-△Vpr infection, to investigate the role of Vpr in DNA methylation variation. Besides, we conducted an analysis on the distinctive features of Vpr-induced differentially methylated cytosines (DMCs), while also investigating the correlation between DNA methylation of histones and Vpr. The findings of this research endeavor will significantly enhance our comprehension regarding the impact of HIV-1 Vpr on the DNA methylation of host cells, thereby presenting innovative therapeutic targets for the regulation of HIV progression and management.

## Methods

### Human participants

The use of healthy adult peripheral blood mononuclear cells for the isolation of primary CD4 + T cells was approved by the Institutional Review Board of Shenzhen Blood Center (Shenzhen, Guangdong, China). All participants provided written informed consent to participate in the study and for publication of their experimental data.

### Virus stock production

Pseudotyped viral stocks were produced in HEK293 T cells by co-transfecting 2.2 µg of pCXCR4 HIV-1 envelope-expression vector, 4.4 µg of packaging vector pC-help, and 4.4 µg HIV-1 pseudotyped vector using a polyethylenimine (Invitrogen) transfection system according to the manufacturer’s instructions. The lentivirus for overexpression was produced in HEK293 T cells by co-transfecting 2.2 µg of VSV-G glycoprotein-expression vector, 4.4 µg of packaging vector pC-help, and 4.4 µg of lentivirus vector. Supernatants were harvested after 48 h, centrifuged (10 min, 500 × g, room temperature), and filtered through a 0.45 μm pore size membrane to remove the cell debris. The viruses were concentrated by centrifugation with 25% v/v of 50% polyethylene glycol 6000, 10% v/v of 4 m NaCl and 11% v/v of 4 PBS. Concentrated virions were resuspended in complete medium and stored at -80 °C.

### HIV-1 infection and cell culture

Primary CD4 + T cells were isolated from three HIV-1 naïve individuals. CD4 + T cells were co-stimulated with anti-CD3 and anti-CD28 antibodies (BioLegend) for three days. Activated CD4 + T cells were infected with pseudotyped viral stocks at a multiplicity of infection of 0.05–0.1. All infections were performed by centrifuging the target cells with the virus at 1,200 × g for 2 h. The infected cells were maintained in STCM (super T cell medium) for 3 days or 7 days.

### Genomic DNA isolation and WGBS library construction

At 3 days post-infection and 7 days post-infection, each DNA samples were extracted using the TIANamp genomic DNA kit (TIAN GEN, China) and three individuals were mixed in equal proportions. For the construction of the typical WGBS library, 1 µg sample genome DNA mixed with unmethylated lambda DNA was fragmented using a Bioruptor system to a mean size of approximately 250 bp. After fragmentation, the purified randomly fragmented DNA was treated with a mix of T4 DNA polymerase, Klenow fragment and T4 polynucleotide kinase to repair, blunt, and phosphorylate the ends. The blunt DNA fragments were subsequently 3’ adenylated using the Klenow fragment (3’–5’ exo-) and ligated to adaptors synthesized with 5’-methylcytosine instead of cytosine using T4 DNA ligase. After each step, DNA was purified using a QIAquick PCR Purification Kit (Qiagen). Next, a ZYMO EZ DNA Methylation-Gold Kit™ was used according to the manufacturer’s instructions to convert unmethylated cytosine into uracil. Finally, PCR was carried out in a final reaction volume of 50 µL, consisting of 20 µL adapter ligated DNA, 4 µL 2.5mm dNTP, 5 µL 10 × buffer, 0.5 µL JumpStart™ Taq DNA Polymerase, 2 µL PCR primers and 18.5 µL water and the following thermal cycling program: 94 °C for 1 min, 10 cycles of 94 °C for 10 s, 62 °C for 30 s, 72 °C for 30 s then prolonged with 5 min at 72 °C and the products held at 12 °C. PCR products were purified using the QIAquick PCR Purification Kit (Qiagen, Hilden, Germany). Before analysis using the Illumina sequencing platform (Illumina Novaseq 6000 paired 150), the purified library was analyzed using a Bioanalyzer analysis system (Agilent, Santa Clara, CA, USA) and quantified by real-time PCR.

### DNA-seq data analysis

Adaptor sequences, contaminants, and low-quality reads were initially removed from the raw data following the delivery of the sequencing data. The reference genome was then mapped using the clean data with BSMAP [38], and a sufficient amount of clean data was ensured. The reference genome utilized in this study was based on prior research [39]. The alignment underwent a quality check. The methylation information for cytosine throughout the whole genome was subsequently obtained using the uniquely mapped data. The cytosine data were then employed for generic and customized bioinformatics analyses.

### Sequence alignment and detection of cytosine methylation levels

The cleaned reads were mapped back to the reference genome using BSMAP software version 2.90. The parameter settings used were “-n 1 -v 0.08 -g 1 -p 48”. The methylation ratios were extracted from the BSMAP output using a Python script (methratio.py) distributed with the BSMAP package. Briefly, the methylation level was calculated based on the methylated cytosine (mC) percentage in the whole genome as follows: site methylation level = 100 × (number of sequences with methylated cytosines (mC)/total number of valid sequences). DMCs were detected using MethylKit (v1.20) in de-novo mode among CpG sites with at least 5 × coverage. The parameter settings used were “--mincov 5 --m_cutoff 0.1 --p_cutoff 0.05”. The genome GO and KEGG annotation was performed using emapper based on the EGGNOG database. Related genes were subjected to GO and KEGG enrichment analyses using Allenricher (v1.0).

### Differentially methylated region (DMRs) detection

Windows with at least five CG sites, a twofold change in methylation level, and Fisher test *P* < 0.05 were selected to identify putative differentially methylated regions (DMRs) between the control and other groups. Neither group should be hypomethylated during DMR discovery. If the genomic area from the beginning of an upstream DMR to the end of a downstream DMR displayed twofold methylation level variations between Con and other groups with a *P* < 0.05, then the two neighboring DMRs were deemed interdependent and combined into one continuous DMR. The two DMRs were considered independent in all other respects. The final dataset of DMRs comprised those of DMRs independent of one another after repeatedly combining the interdependent DMRs.

### Function enrichment of DMR-related genes

Gene Ontology (GO) enrichment histogram of DMR-related genes can visually reflect the number distribution of DMR-related genes in BP (biological process), CC (cell component) and MF (molecular function). Kyoto Encyclopedia of Genes and Genomes (KEGG) is the main public database on the pathway. ClusterProfiler package in R software was used to investigate GO and KEGG enrichment analysis. *P* < 0.05 was used for the cut-off criterion.

### Quantitative PCR (qPCR)

The cells were then incubated with different compounds for different times. Total RNA was extracted using the TRIzol reagent (Thermo Fisher, Cat# 15,596,018). Reverse transcription of RNA to cDNA was performed using SuperScript IV system-controlled response (Thermo Fisher Scientific). qPCR was performed using SYBR qPCR Master Mix (Vazyme, Nanjing, China) on the QuantStudio 3 PCR system (Thermo Fisher Scientific) using a standard two-step procedure (denaturation at 95 ℃ for 10 s, annealing at 60 ℃ for 30 s; 40 cycles). The qPCR primer sequences are listed in Table 1. The glyceraldehyde-3-phosphate dehydrogenase (GAPDH) gene was used as the reference gene, and the relative expression of mRNAs was analyzed using 2- ΔΔCT.

**Table 1:** RT-qPCR primer sequences

Primer name	Sequence (5’-3’)
H1-2-F	CCGCCTCTAAAGAGCGTAGC
H1-2-R	AGACCAAGTTTGATACGGCTG
H2AC6-F	CTCGCGCCAAAGCGAAATC
H2AC6-R	CTGCGTAGTTGCCTTTACGGA
H2AC8-F	CTACTCCGAACGAGTCGGG
H2AC8-R	GATGGTCACGCGACCTAGAAG
H2BC5-F	GGGCATCATGAACTCGTTCG
H2BC5-R	ACTTGGAGCTGGTGTACTTGG
H3C4-F	CCATTCCAGCGTCTAGTCCG
H3C4-R	TCTGAAAACGCAGATCAGTCTTG

### Western blotting

After treatment, cells were lysed by Radio Immunoprecipitation Assay (RIPA) lysis buffer, incubated on ice for 30 min, followed by centrifugation at 12,000 × g for 10 min at 4 ℃. The supernatant was collected as the whole protein extract. The protein extract was denatured by adding NuPAGE running buffer (Thermo Fisher Scientific, Cat# NP0001) and heating up at 100 ℃ for 10 min. Protein samples were stored at − 80 ℃ or used directly for western blotting. Total proteins were electrophoresed on an SDS-PAGE gel for 1.5 h to separate the proteins, which were then transferred onto a polyvinylidene fluoride (PVDF) membrane (Bio-Rad, Cat# 1,620,177). They were then co-incubated with primary antibodies and incubated with secondary antibodies conjugated to horseradish peroxidase (HRP). Antibodies were diluted with 5% bovine serum albumin (BSA). Primary antibodies against DNMT1 (24206-1-AP) and DNMT3A (20954-1-AP), were purchased from proteintech Group (Wuhan, China). Primary antibodies CTCF (ab128873), PAX5 (ab109443) and GAPDH (ab181602) were purchased from Abcam (Cambridge, UK). HRP-labeled Goat Anti-Mouse IgG (H + L) (A0216) and HRP-labeled Goat Anti-Rabbit IgG (H + L) (A0208) were purchased from Beyotime (Guangzhou, China). The HRP signal was detected using ChemiDoc Touch (Bio-Rad).

### Statistical analysis

All experiments were repeated at least thrice. Statistics were performed with GraphPad Prism 8 version 8.0 (GraphPad Software, Inc., San Diego, CA, USA), and all data are presented as mean values ± SD. *p*-values were calculated based on the Student’s t-test using IBM SPSS statistics for Windows, version 20.0 (IBM Corp., Armonk, NY, USA).

## Results

### HIV-1 Vpr induces DNA methylation changes in primary CD4 + T cells

**Fig. 1:**
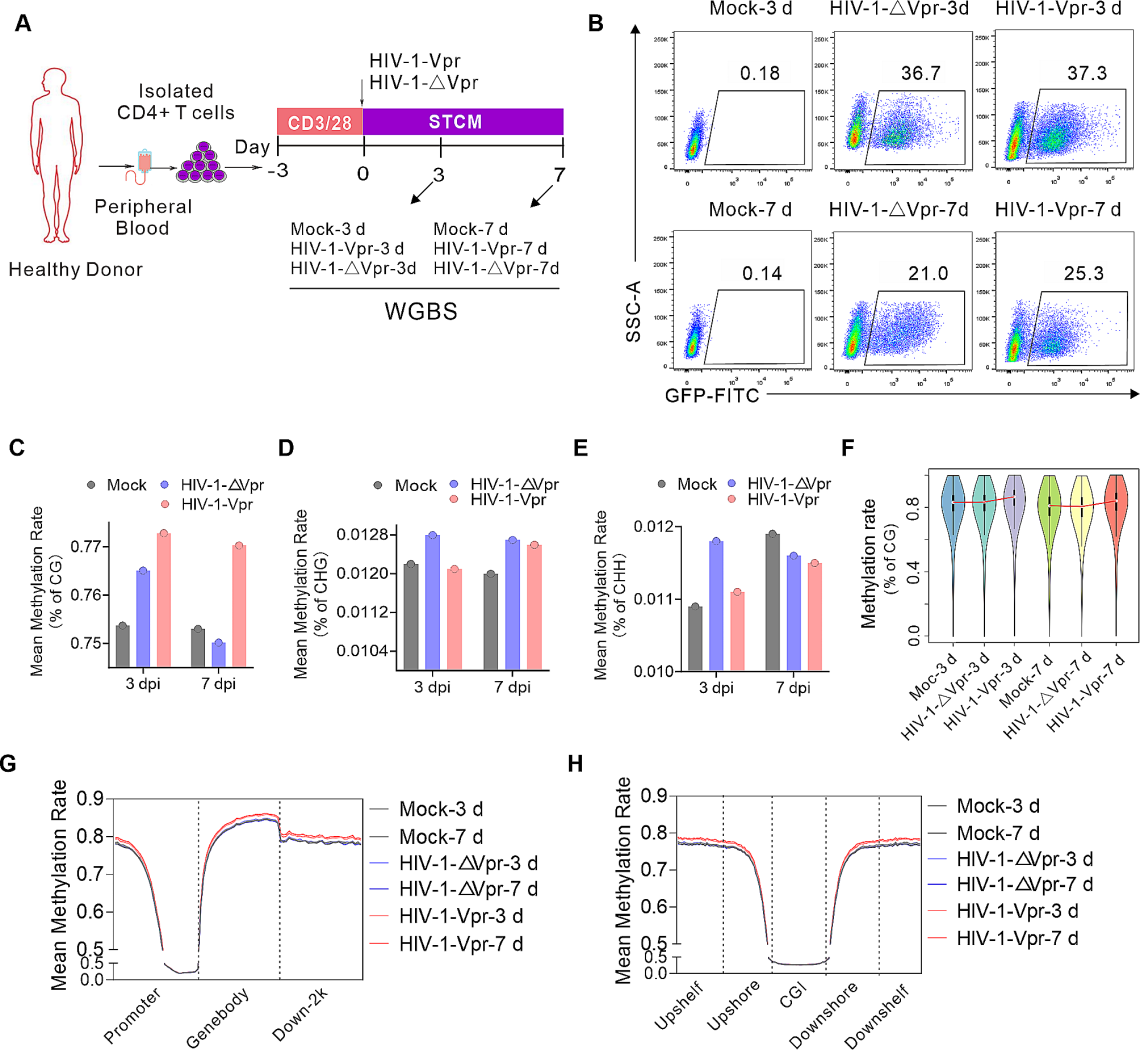
Landscape of Vpr-induced DNA methylation. (**A**) The schematic depicts the experimental design employed in the Whole Genome Bisulfite Sequencing (WGBS) study. (**B**) Flow cytometry plots are presented as representatives, illustrating the HIV-1 infection of primary CD4 + T cells in the context of WGBS. (**C**–**E**) The methylation levels across the genome are displayed for CG (**C**), CHG (**D**), and CHH (**E**) contexts. (**F**) A violin plot is utilized to showcase the overall distribution of methylation levels, with a bin size of 10 kb. (**G**, **H**) The mean DNA methylation levels are computed based on elemental (**G**) and CGI (**H**) patterns.

Activated CD4 + T cells were infected with either HIV-1-Vpr or HIV-1-△Vpr. The infection rates remained consistent in both groups on day 3 or 7 post-infection, and all samples were collected for subsequent analysis using whole-genome bisulfite sequencing (WGBS) (Fig. 1A, B). A total of 80 G clean data was generated for each group, and after data filtering, the mapped reads exhibited rates ranging from 73.25 to 77.18% (Table S1). Notably, DNA methylation was observed in three sequence contexts, namely CG, CHG, and CHH (Table S2). The distribution of the three types of methylation exhibited consistency across all groups, with mCG accounting for approximately 75.4–77.28%, mCHG accounting for 1.20–1.28%, and mCHH accounting for 1.0–1.18% (Table S2). Our findings indicate that Vpr solely augmented the average methylation rate in CG, while no effect was observed in CHG or CHH (Fig. 1C-E). Although minimal variation was observed in the methylation patterns of promoter regions and CpG islands (CGI), Vpr heightened the levels of DNA methylation in the gene body, 2 kb downstream of the transcription start site (TSS), and CGI shelves (Fig. 1F-H). Together, there data indicated that HIV-1 Vpr can induce DNA methylation changes, especially CG type DNA methylation in primary CD4 + T cells.

**HIV-1 Vpr induces extensive DNA hypermethylation.**

**Fig. 2:**
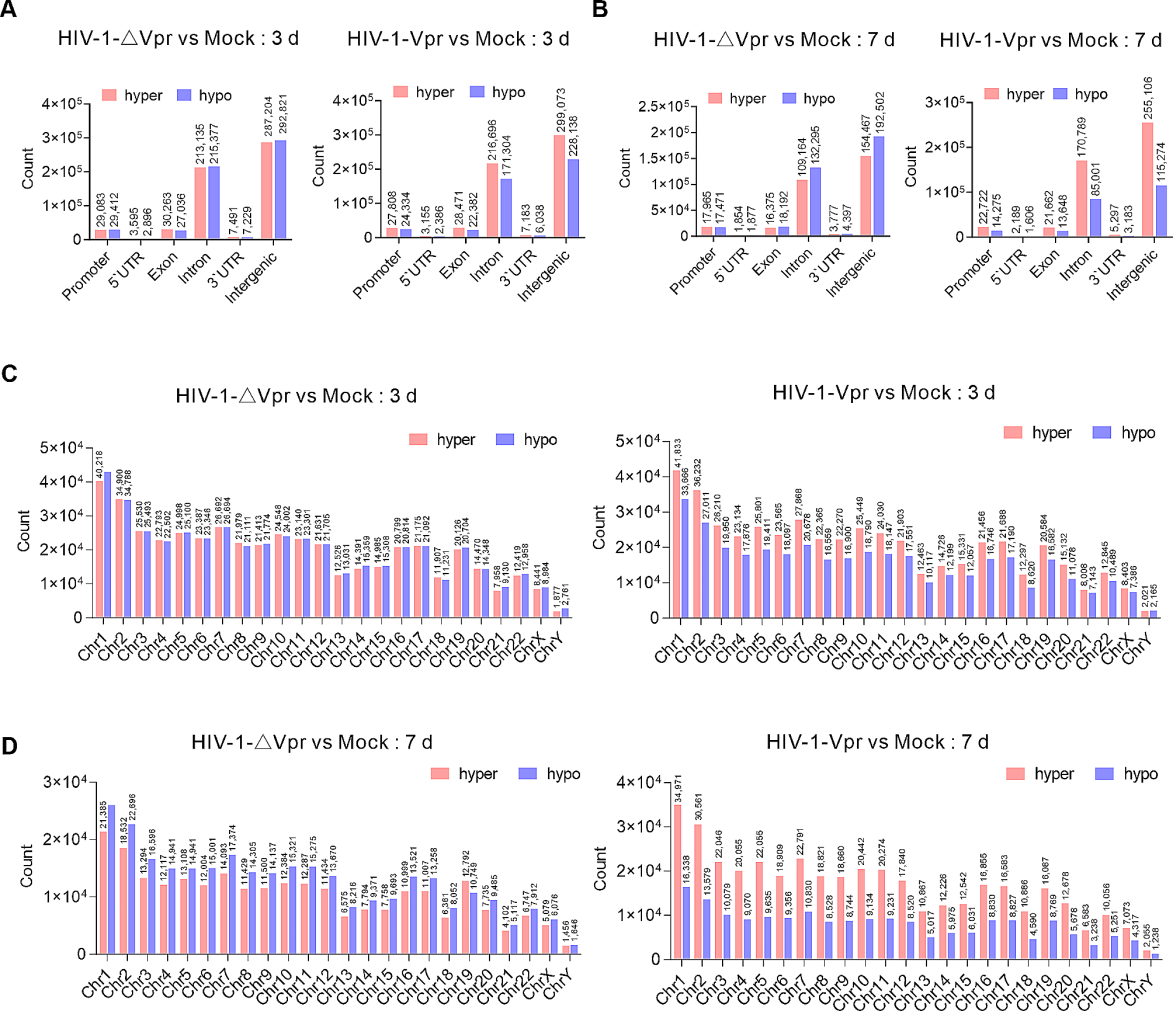
HIV-1 Vpr induce extensive DNA hypermethylation. (**A**) Genomic characteristics of differentially methylated cytosines (DMCs) in HIV-1-∆Vpr infection and HIV-1-Vpr infection on day 3 post-infection. (**B**) Genomic features of differentially methylated cytosines (DMCs) in HIV-1-∆Vpr infection and HIV-1-Vpr infection on day 7 post-infection. (**C**) The distribution of DMCs on each chromosome during HIV-1-∆Vpr or HIV-1-Vpr infection on day 3 post-infection. (**D**) The distribution of DMCs on each chromosome during HIV-1-∆Vpr or HIV-1-Vpr infection on day 7 post-infection.

In order to further investigate the distribution of methylation levels in various transcriptional elements, we categorized mC into specific gene features, including promoter, 5`UTR, exon, intron, 3`UTR, and intergenic regions. Analysis of the distribution of genomic features revealed that there were no significant differences in genome-wide DNA methylation levels on day 3 after HIV-1-∆Vpr infection. However, on day 7, there was a slight increase in the proportion of hypomethylated cytosines (Fig. 2A, B). However, the incidence of hypermethylated cytosines exhibited a substantial increase upon HIV-1-Vpr infection, predominantly localized within the gene body and intergenic regions (Fig. 2A, B), whereas changes in the level of each chromosome remained consistent (Fig. 2C, D). These findings provide evidence that primary CD4 + T lymphocytes exposed to Vpr experienced notable DNA hypermethylation, particularly within the gene body and intergenic regions, in the context of HIV-1 infection.

Vpr orchestrates the DNA methylation of lymphocyte differentiation-related genes.

**Fig. 3:**
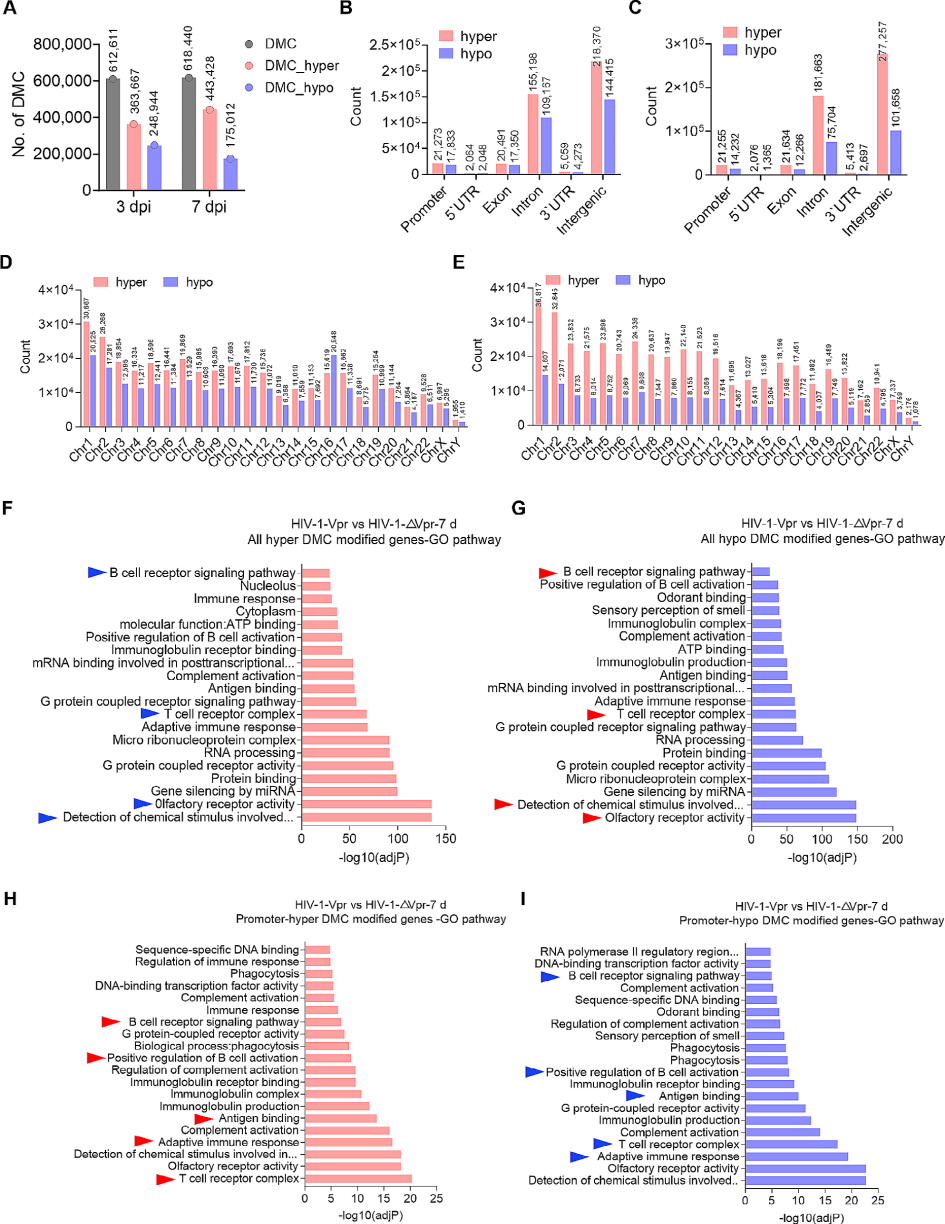
Vpr-induced DNA methylation changes mainly distributed in immune-related pathways. (**A**) The quantity of differentially methylated cytosines (DMCs) specifically induced by Vpr. (**B**, **C**) The genomic characteristics of DMCs specifically induced by Vpr on day 3 (**B**) and day 7 post-infection (**C**). (**D**, **E**) The distribution of DMCs specifically induced by Vpr on each chromosome on day 3 (**D**) and day 7 post-infection (**E**). (**F**, **G**) The Gene Ontology (GO) enrichment analysis of genes associated with all-hypermethylated (**F**) or hypomethylated (**G**) DMCs. (**H**, **I**) The GO enrichment analysis of genes associated with promoter-hypermethylated (**H**) or hypomethylated (**I**) DMCs.

A comparison between HIV-1-Vpr infection and HIV-1-∆Vpr infection yielded a total of 612,611 differentially methylated cytosines (DMCs). Among these DMCs, 363,667 were hypermethylated cytosines and 248,944 were hypomethylated cytosines. These changes were predominantly observed in genosome and intergenic regions on day 3 post-infection, as depicted in Fig. 3A-C. Notably, Vpr-induced infection on day 7 resulted in a higher number of hypermethylated cytosines, as shown in Fig. 3A-C. The methylation changes in each chromosome remained constant, except for chromosome 16 (Fig. 3D, E). GO analysis was conducted to explore the functional significance of genes modified by DMC, revealing that both hypermethylation and hypomethylation modifications were found to be enriched in specific pathways, including olfactory receptor activity, the B cell receptor signaling pathway, and the T cell receptor complex (Fig. 3F, G). Additionally, promoter-DMC-modified genes were predominantly associated with the T cell receptor complex, olfactory receptor activity, and B cell receptor signaling pathways (Fig. 3H, I), which are known to be epigenetic markers associated with gene silencing [40]. The findings suggest that methylation modification on either the gene body or promoter leads to the clustering of Vpr-induced hypermethylated cytosine-modified genes and Vpr-induced hypomethylated cytosine-modified genes into highly similar pathways.

**Fig. 4:**
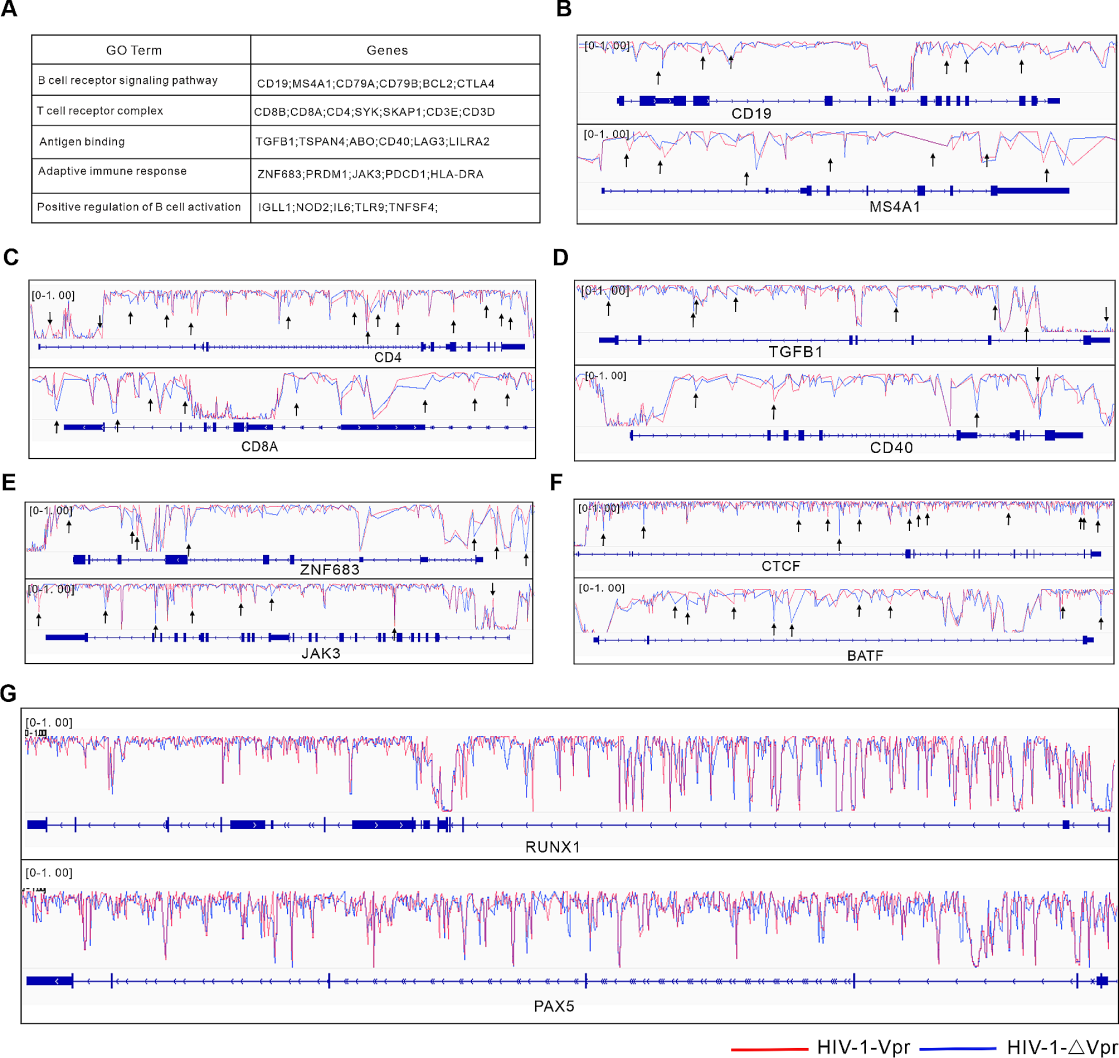
Vpr induces DNA methylation changes in lymphocyte differentiation-related genes. (**A**) Signature genes of the main GO enrichment pathways of Vpr-induced DMCs. (**B**) The IGV genome browser was used to track whole-genome bisulfite sequencing (WGBS) data at the CD19 or MS4A1 genes in HIV-1-∆Vpr and HIV-1-Vpr infections. (**C**) The IGV genome browser was utilized to observe WGBS tracks at the CD4 or CD8A genes in HIV-1-∆Vpr and HIV-1-Vpr infections. (**D**) WGBS data at the TGFB1 or CD40 genes in HIV-1-∆Vpr and HIV-1-Vpr infections were visualized using the IGV genome browser tracks. (**E**) The IGV genome browser was employed to examine WGBS tracks at the ZNF683 or JAK3 genes in HIV-1-∆Vpr and HIV-1-Vpr infections. (**F**) WGBS data at the CTCF or BATF genes in HIV-1-∆Vpr and HIV-1-Vpr infections were analyzed using the IGV genome browser tracks. (**G**) The IGV genome browser was used to track WGBS data at the RUNX1 or PAX5 genes in HIV-1-∆Vpr and HIV-1-Vpr infections. Black arrows indicate DMCs.

Figure 4 A illustrates the presence of specific Vpr-induced DMC-modified genes that exhibit enrichment in T/B cell-related pathways, including CD19 and MS4A1 (B cell biomarkers), CD4 and CD8A (T cell biomarkers), TGFB1 and CD40 (antigen binding), and ZNF683 and JAK3 (adaptive immune response) (Fig. 4B-E). Furthermore, Vpr perturbed the DNA methylation patterns of BATF, CTCF, RUNX1, and PAX5, which are crucial transcriptional regulators involved in lymphocyte differentiation (Fig. 4F, G) [41–44]. These findings collectively provide evidence that Vpr induces alterations in the DNA methylation of genes associated with lymphocyte differentiation.

**Fig. 5:**
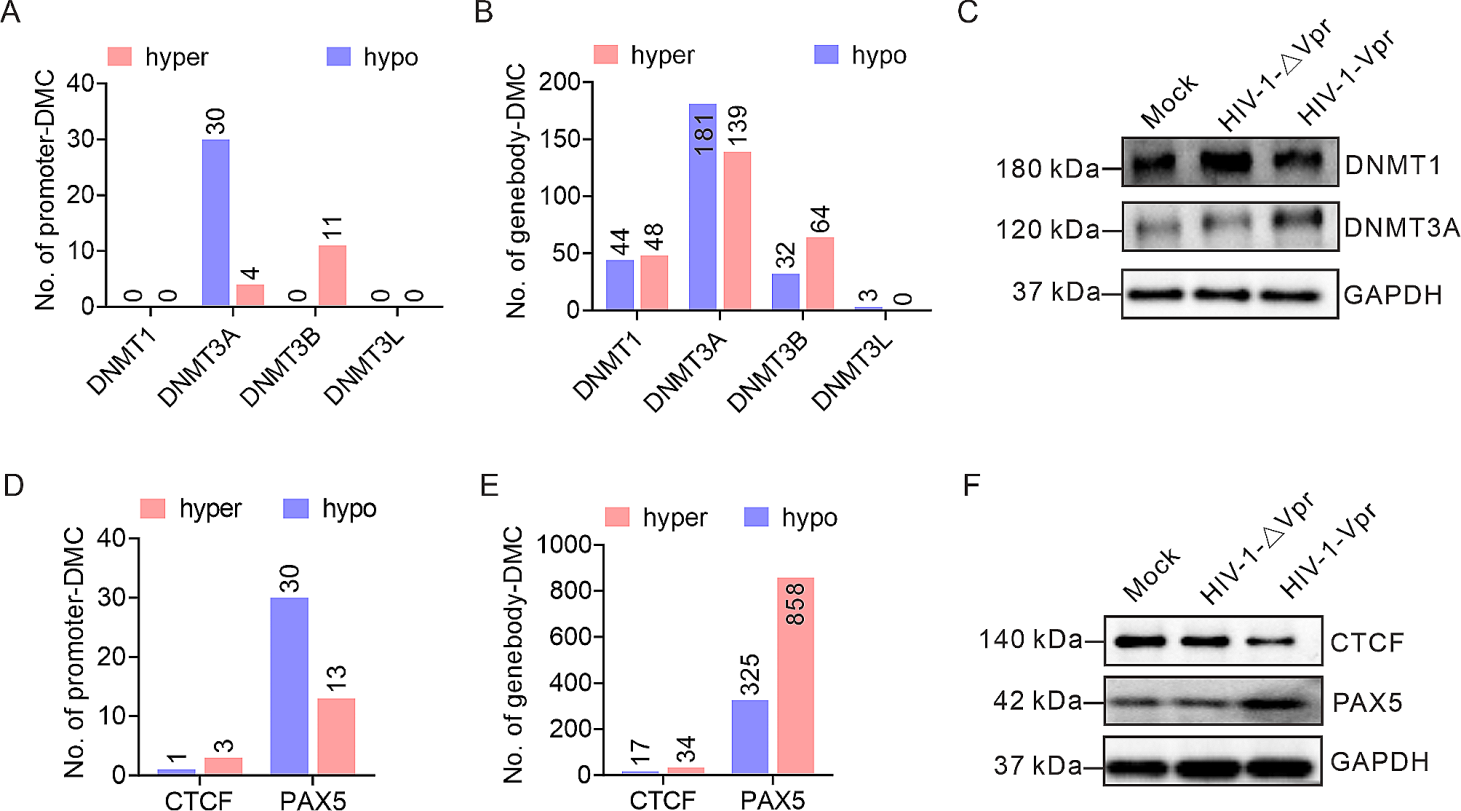
The impact of Vpr on DNA methylation of host cell is verified by western blot. (**A**, **B**) The effect of Vpr on differentially methylated cytosines (DMCs) in the promoter (**A**) or gene-body (**B**) region of DNMTs. (**C**) The effect of Vpr on protein levels of DNMT1 and DNMT3A. (**D**, **E**) The effect of Vpr on DMCs in the promoter (**A**) or gene-body (**B**) region of CTCF and PAX5. (**F**) The effect of Vpr on protein levels of CTCF and PAX5.

DNA methylation is orchestrated by a family of DNMTs, notably DNMT1 and DNMT3A [19, 20]. Our investigation revealed that Vpr induces demethylation in the promoter region of DNMT3A, while DNMT1 remains unchanged (Fig. 5A, B). Our western blot analysis demonstrated that Vpr enhances the expression of DNMT3A without affecting DNMT1 levels (Fig. 5C), corroborating our sequencing data. CTCF, a pivotal transcription factor with conserved functions in cell type-specific genome organization [43]. Our results showed that Vpr induced hypermethylation in the promoter region of CTCF (Fig. 5D), coupled with decreased CTCF expression (Fig. 5F). Furthermore, Vpr exerts a substantial influence on the DNA methylation status of PAX5, with 30 hypomethylated promoter-DMCs and 858 hypermethylated gene-body-DMCs induced by Vpr (Fig. 5E). Intriguingly, western blot data indicated an increase in PAX5 expression upon Vpr exposure (Fig. 5F). Collectively, these results suggested that HIV-1 Vpr alters DNA methylation of lymphocyte differentiation-related genes by orchestrating the expression of DNMTs.

### HIV-1 vpr alters differentially methylated regions (DMRs) in primary CD4 + T cells

**Fig. 6:**
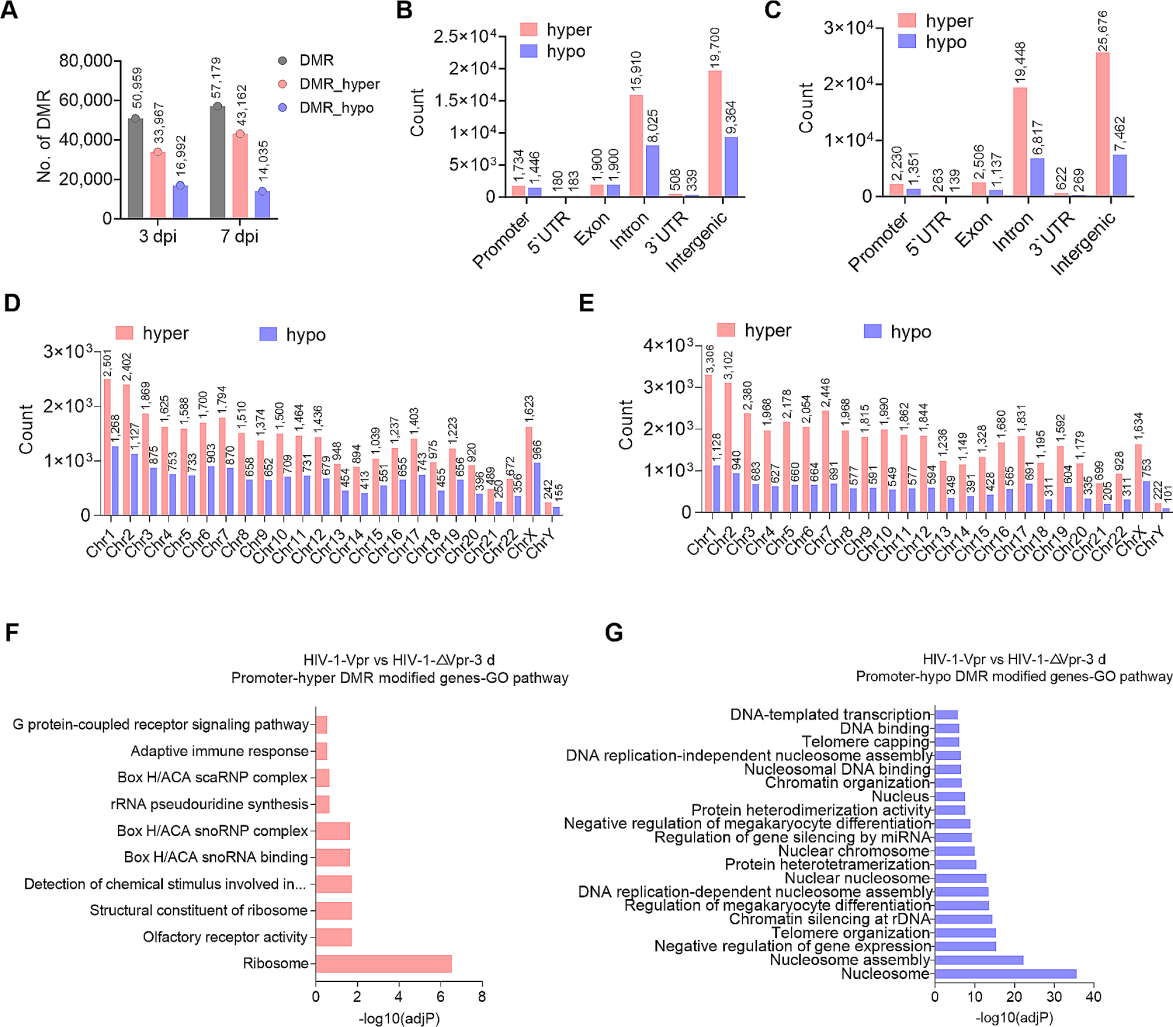
Vpr alters differentially methylated regions (DMRs) in primary CD4 + T cells. (**A**) The quantity of differentially methylated regions (DMRs) induced by Vpr. (**B**, **C**) The genomic characteristics of Vpr-induced DMRs on the third day after infection (**B**) and on the seventh day after infection (**C**). (**D**, **E**) The distribution of Vpr-induced DMRs on each chromosome on the third day after infection (**D**) and on the seventh day after infection (**E**). (**F**, **G**) The enrichment of gene ontology (GO) in promoter-hyper (**F**) or hypo (**G**) DMCs modified-associated genes.

In order to further investigate the disparity in DNA methylation between the HIV-1-Vpr group and the HIV-1-∆Vpr group, we conducted a quantitative analysis of differentially methylated regions (DMRs), which are distinct DNA segments exhibiting varying methylation patterns, across the various groups. Our findings revealed the presence of 33,967 hyper-DMRs and 16,992 hypo-DMRs on day 3 post-infection, as well as 43,162 hyper-DMRs and 14,035 hypo-DMRs on day 7 post-infection in the HIV-1-Vpr group compared to the HIV-1-∆Vpr group (Fig. 6A). Additionally, we conducted a comprehensive analysis of the genomic characteristics of these DMRs and observed that the majority of DMRs were located within intronic and intergenic regions. Furthermore, it was noted that the number of hyper-DMRs significantly exceeded that of hypo-DMRs in each genomic element (Fig. 6B, C). In each chromosome, there was a significantly greater number of hyper-DMRs compared to hypo-DMRs (Fig. 6D, E). To further explore the functional role of DMR-modified genes, a GO analysis was conducted. It was observed that promoter-hyper-DMRs modified genes were predominantly enriched in ribosome and ribosome-related pathways (Fig. 6F). Surprisingly, promoter-hypo-DMRs modified genes were found to be highly enriched in nucleosomes and nucleosome assembly related pathways (Fig. 6G). These findings suggest that HIV-1 Vpr specifically induces a higher number of hyper-DMRs in primary CD4 + T cells.

### HIV-1 vpr drives loss of histone DNA methylation

**Fig. 7:**
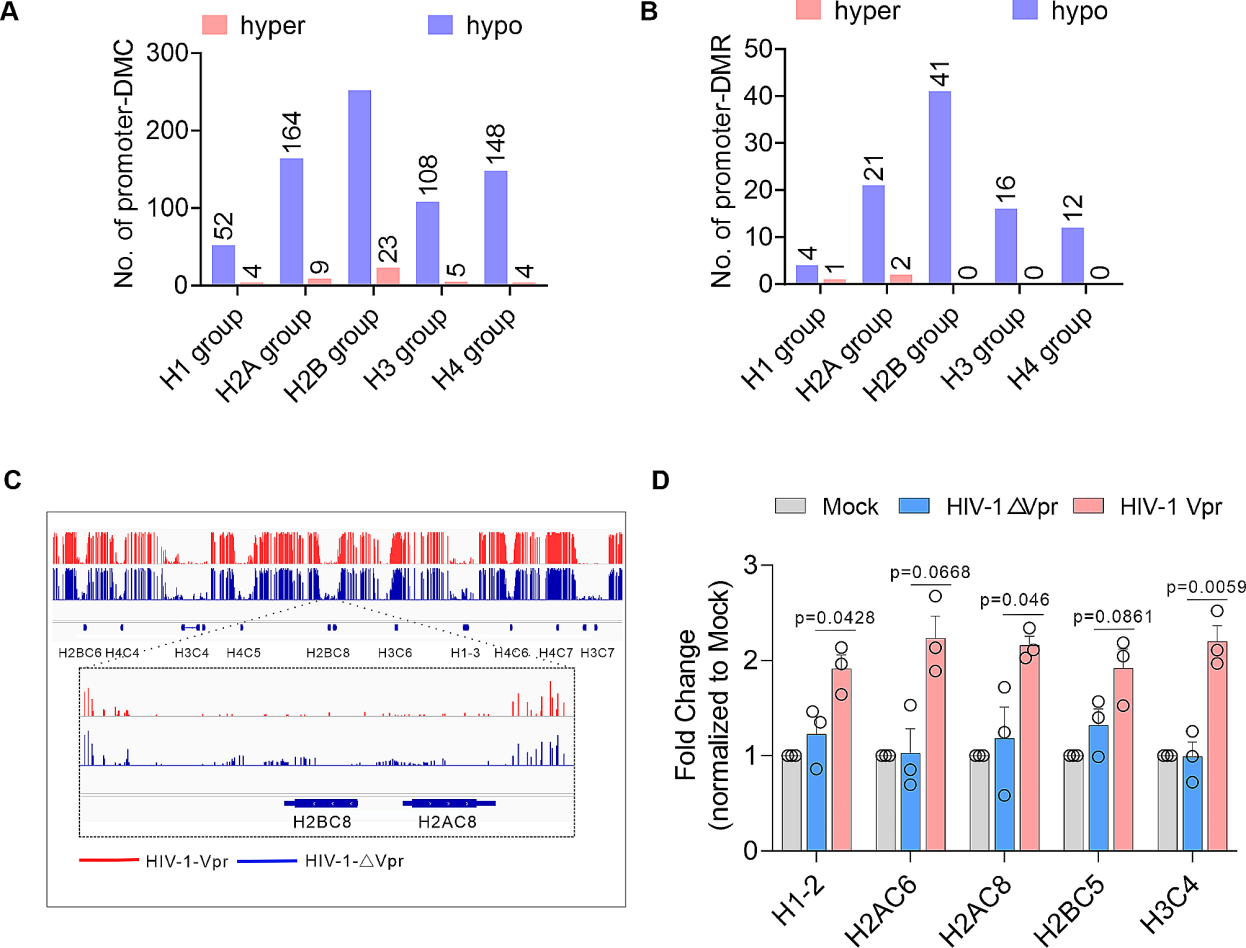
Vpr induces loss of histone DNA methylation. (**A**, **B**) The effect of Vpr on histone DNA methylation. Number of differentially methylated cytosines (DMCs) (**A**) or differentially methylated regions (DMRs). (**C**) Example of genome browser tracks of histone DNA methylation profiles in HIV-1-△Vpr and HIV-1-Vpr infection. (**D**) The effect of Vpr on the transcription of histone genes through real-time quantitative RCR. Data are presented as means ± SD for three technical replicates. *P* values were calculated using an unpaired *t*-test.

Although Vpr exhibits a specific ability to induce a greater number of hyper-differentially methylated regions (DMRs) in primary CD4 + T cells, the genes that are modified by Vpr-induced promoter hypo-DMRs are predominantly associated with nucleosomes and nucleosome assembly pathways (Fig. 6G). In order to further investigate the potential loss of histone DNA methylation induced by Vpr, we conducted an analysis of methylation levels in the promoter region of all histone genes (Table S3). Our findings indicate that Vpr is capable of inducing demethylation in the promoter region of all histone genes (Fig. 7A, B). This conclusion is further supported by the results obtained from IGV genome browser analysis of histone DNA methylation profiles, which confirm that Vpr can indeed reduce DNA methylation levels of histone genes (Fig. 7C). Furthermore, we found that Vpr can indeed promote the transcription of histone genes through real-time quantitative RCR (Fig. 7D). Together, the results suggested that HIV-1 Vpr plays an important role in regulating histone gene expression.

## Discussion

In this study, we conducted an assessment of the impact of Vpr on DNA methylation in primary CD4 + T cells during HIV-1 infection, utilizing WGBS. Our findings reveal that Vpr has a significant influence on DNA methylation, particularly in CG type DNA methylation within primary CD4 + T cells. A total of 612,611 differentially methylated cytosines (DMCs) were identified between the HIV-1-Vpr group and the HIV-1-∆Vpr group, with Vpr inducing a higher number of hyper-DMCs compared to hypo-DMCs, particularly on day 7 post-infection. Moreover, Vpr augmented the average methylation rate in gene bodies and intergenic regions. The prevailing form of DNA methylation observed is commonly referred to as “gene body methylation.” It is noteworthy that the influence of promoter methylation on gene expression differs from that of gene body methylation [45–48]. Promoter methylation is commonly associated with transcriptional repression, whereas gene body methylation is frequently associated with active transcription in humans and other animals [47, 48]. Consequently, it can be inferred that Vpr enhances gene expression by augmenting gene body DNA methylation.

Genes modified by DMCs in both the gene body and promoter regions were significantly enriched in olfactory receptor activity and the detection of chemical stimuli involved in the sensory perception of smell. Epigenetic modification of olfactory receptor genes plays a significant role in the development of neurons [49–51]. Olfactory dysfunction is one of the deficiencies in people living with HIV-1 [52, 53], impaired olfactory recognition may be a marker of early central nervous system (CNS) damage in HIV-1 infection [51]. Vpr may lead to decreased olfactory receptor activity and increased olfactory threshold through methylation modification of host olfactory receptor activity and chemically stimuli-related gene regions involved in olfactory perception.

Furthermore, Vpr induces modifications in DMC genes that are involved in immune-related pathways, such as the B cell receptor signaling pathway, T cell receptor complex, adaptive immune response, and antigen binding. Within these pathways, the methylation of certain genes related to lymphocyte differentiation is regulated differently, including lymphocyte differentiation transcription factors (BATF, CTCF, RUNX1, and PAX5) [41, 42, 44, 54, 55], T cell biomarkers (CD4 and CD8A), and B cell biomarkers (CD19 and MS4A1). The differentiation and maturation of T cells and B cells is a multifaceted process influenced by intricate cellular decisions involving transcription factor expression and epigenetic regulators that modify the epigenomic landscape [56, 57]. DNA methylation is a key player in directing the differentiation trajectory of T cell lineage during T cell development [58, 59]. Similarly, DNA methylation not only governs the complex epigenetic characteristics of regulatory elements in B cell differentiation and maturation, but is also prevalent in mature B cells [60, 61]. Our findings collectively demonstrate that Vpr affects the development and differentiation of T cells and B cells by altering the DNA methylation of genes related to lymphocyte differentiation.

Eukaryotic chromatin is organized by nucleosomes, nucleosomes are formed from an octameric core particle of two molecules each of the histones H2A, H2B, H3 and H4, which wraps 147 bp of DNA [62]. The association of nucleosomes with most genomic DNA prevents initiation from cryptic promoters. The nucleosome thus serves not only as a general gene repressor, but also as a repressor of all transcription (genic, intragenic, and intergenic) [63]. Histone marks, including histone acetylation, methylation, ubiquitylation, and phosphorylation were very common and crucial for regulating gene transcription, but there are few reports on DNA methylation modification of histone genes themselves. In our investigation, we observed a strong association between Vpr-induced promoter-hypo-DMRs-modified genes and pathways related to nucleosomes and nucleosome assembly. Additionally, we discovered that Vpr extensively reduces the DNA methylation of histone genes. As we all know that demethylation of the promoter region of a gene will promote the expression of the gene, so HIV-1 Vpr can increase the expression of histone genes by DNA demethylation, and may lead to transcriptional repression of host cell, as previously reported [35–36], which may indirectly also contribute towards G2 arrest by Vpr [64–66]. Together, these findings suggest that Vpr may play a role in the transcriptomic remodeling of CD4 + T cells during HIV-1 infection by regulating the DNA methylation of histones.

## Conclusions

This study elucidated the influence of Vpr on the global methylation patterns of primary CD4 + T cells during HIV-1 infection. Our findings provide evidence that Vpr triggers substantial alterations in DNA methylation, particularly within the promoter and gene body regions, which play a crucial role in regulating the expression of immune-related pathway genes and olfactory receptor activity. Additionally, Vpr exhibits a specific ability to decrease methylation levels of histone genes. Collectively, these results underscore the noteworthy role of Vpr in modulating transcriptional control through the induction of DNA methylation variations. Further research is warranted to gain a comprehensive understanding of the specific mechanisms by which Vpr governs DNA methylation, with particular emphasis on its regulation of histone gene methylation.

### Electronic supplementary material

Below is the link to the electronic supplementary material.

Supplementary Material 1

Supplementary Material 2

Supplementary Material 3

## Data Availability

The original data files of WGBS are available in Genome Sequence Archive at https://ngdc.cncb.ac.cn/gsa-human/, reference number HRA005936. The data deposited and made public are compliant with the regulations of the Ministry of Science and Technology of China. Other data are available from the corresponding author upon reasonable request.

## Abbreviations

BP: biological process
CC: cell component
CGI: CpG islands
DNMTs: DNA methyltransferases
DMCs: differentially methylated cytosines
DMRs: differentially methylated regions
GO: Gene Ontology
KEGG: Kyoto Encyclopedia of Genes and Genomes
MeCP2: Methyl CpG-binding protein-2
MF: molecular function
STCM: super T cell medium
TSS: transcription start site
Vpr: viral protein r
WGBS: whole-genome bisulfite sequencing
